# Recent Changes in Sexually Transmitted Infection in Korea: A Population-Based Analysis

**DOI:** 10.3390/jcm14145145

**Published:** 2025-07-20

**Authors:** Jae Yen Song, Kang Seob Kim, Chang Hee Han, Sangrak Bae

**Affiliations:** 1 Department of Obstetrics and Gynecology, Seoul St. Mary’s Hospital, College of Medicine, The Catholic University of Korea, Seoul 06591, Republic of Korea; jaeyen77@nate.com; 2 Department of Urology, Uijeongbu St. Mary’s Hospital, College of Medicine, The Catholic University of Korea, Seoul 11765, Republic of Korea; prodigy81@catholic.ac.kr (K.S.K.); urohan@catholic.ac.kr (C.H.H.)

**Keywords:** sexually transmitted infections, STI, sexually transmitted diseases, STD, prevalence, incidence

## Abstract

**Background**: The objective of this study is to investigate the prevalence and epidemiological changes of major sexually transmitted infections (STIs) in Korea over the past decade. **Methods**: From 2010 to 2021, patients diagnosed with STIs based on ICD-10 codes were analyzed using Korean Health insurance data. The analysis included the number of patients, prevalence, and age-specific prevalence (in 5-year intervals) over this period. We examined changes in disease patterns over time by analyzing the annual trends and age-specific prevalence of bacterial STIs such as chlamydia, mycoplasma, gonorrhea, and syphilis; viral STIs such as genital herpes, human papillomavirus (HPV), and human immunodeficiency virus (HIV); and other infections including scabies, pubic lice, and trichomoniasis. **Results**: In 2010, the STI with the highest prevalence due to an infectious pathogen was trichomoniasis (256.65/100,000), while latent syphilis had the lowest prevalence (5.29/100,000). In 2021, the STI with the highest prevalence was genital herpes (254.54 per 100,000 persons), and latent syphilis continued to have the lowest prevalence. Bacterial STIs showed a decreasing trend. Viral STIs showed a continuous increase throughout the study period, with anogenital warts (AGW) having the highest rate of increase. Other infections showed a decreasing trend. HIV and AGW in men showed a rapid increase. Gender differences varied depending on the disease. **Conclusions**: While bacterial STIs have gradually declined, viral STIs have continued to increase during last decade. The characteristics of each pathogen vary according to age and gender, necessitating the establishment of risk groups for each pathogen and the development of prevention policies accordingly.

## 1. Introduction

Sexually transmitted infections (STIs), also known as venereal diseases or sexually transmitted diseases, refer to infections that are transmitted through sexual contact [1]. STIs represent a significant public health concern worldwide, including in South Korea. These infections, caused by a diverse range of pathogens including bacteria, viruses, and protozoa, can lead to severe health complications if left untreated. Moreover, these infections are often associated with significant impairments in quality of life, including adverse effects on emotional health and sexual function [2]. Over the past decade, the landscape of STI prevalence has undergone notable changes, influenced by factors such as evolving sexual behaviors, public health interventions, and advancements in diagnostic technologies [1].

STIs have a long and complex history, deeply intertwined with human society, health, and cultural practices. There was a time when these infections were called venereal diseases (or diseases of Venus), and outbreaks occurred in medieval Europe. In the 20th century, they became known as sexually transmitted diseases. Since the 2000s, with the increasing recognition of asymptomatic infections, the term sexually transmitted infection has also come into use. The understanding and management of these infections have evolved significantly over the centuries, shaped by advancements in medical science, public health initiatives, and changing social attitudes.

Recent updates to the US Centers for Disease Control and Prevention (CDC) guidelines [3] and recent studies have indicated that in the United States [4], the prevalence of gonorrhea, chlamydia, and syphilis is increasing, while the prevalence of herpes simplex virus (HSV) is decreasing. A study based on WHO statistics indicated that syphilis, chlamydia, trichomoniasis, and genital herpes showed an increasing trend [1]. In addition to the increase in incidence and prevalence, the 2021 CDC guidelines introduced several changes, including concerns about azithromycin resistance, an increase in ceftriaxone dosage, the designation of doxycycline as a first-line treatment, and changes in treatment based on the antibiotic susceptibility of mycoplasma [3]. In Korea, the treatment guideline of STI were also updated, taking into account the 2021 CDC guidelines and adapting them to domestic context [5,6,7,8,9].

Understanding the epidemiological changes in STI prevalence is crucial for developing effective prevention and treatment strategies [10]. However, there has been limited research conducted on the prevalence of STIs in Korea.

This study aims to investigate the prevalence and epidemiological shifts of major STIs in South Korea from January 2010 to December 2021, utilizing comprehensive data from the Health Insurance Review and Assessment Service.

## 2. Materials and Methods

From January 2010 to December 2021, patients diagnosed primarily with STIs were included in the study. Data on these patients were obtained from the Big Data service of the Health Insurance Review and Assessment Service (https://opendata.hira.or.kr/op/opc/olap3thDsInfoTab1.do, accessed on 2 November 2024). The data used in this study are publicly available on the internet and accessible to anyone. The STIs of interest, identified by ICD-10 codes, included chlamydia infection (A56), gonorrhea (A54), genital herpes (A60), mycoplasma infection (A493), AGW (A630), syphilis (primary: A51, latent: A52, tertiary and neurosyphilis: A53), trichomoniasis (A59), HIV infection (B24), pediculosis (Pubic lice, B85.3), scabies (B86), and non-gonococcal urethritis (N34).

Prevalence rates, including overall, gender-specific, and age-specific rates, were extracted and analyzed for these diagnoses. For each disease, the number of patients per year was identified, and prevalence and total patient counts were analyzed based on national population data. Annual population figures by sex and age group were obtained from the Korean Statistical Information Service (KOSIS, https://kosis.kr/index/index.do, accessed on 2 November 2024) The number of cases was extracted using the relevant ICD-10 codes for each year, including total, sex-specific, and five-year age interval breakdowns. Prevalence rates were then calculated based on these population data.

Additionally, based on the type of pathogen, the diagnoses were categorized into bacterial STIs, viral STIs, STIs caused by protozoa and parasites, and non-gonococcal urethritis, which is not associated with a specific pathogen. Bacterial STIs analyzed included mycoplasma, chlamydia, gonorrhea, and early and latent syphilis. Viral STIs included genital HSV, AGW caused by HPV, and HIV infection. Other infections were classified into those caused by protozoa, such as trichomoniasis, and parasites, such as scabies and pediculosis. Additionally, non-gonococcal urethritis, a general diagnosis not associated with a specific pathogen, was also analyzed.

To calculate age-specific and overall prevalence rates, population statistics in 5-year age intervals from Statistics Korea were used. The prevalence rates were calculated using the following formulas:

$$\mathrm{Total}\;\mathrm{prevalence}\;(\mathrm{Per}\;100,000)=\frac{Number\;of\;existing\;cases\;of\;a\;disease}{Total\;population}\times 100,000$$

$$\begin{array}{l} \mathrm{Age}\text{-}\mathrm{specific}\;\mathrm{prevalence}\;(5\text{-}\mathrm{year}\;\mathrm{age}\;\mathrm{interval})\\ \;=\frac{Number\;of\;existing\;cases\;of\;a\;disease\;in\;the\;5\text{-}year\;age\;group}{Total\;population\;of\;the\;5\text{-}year\;age\;group}\times 100,000 \end{array}$$

Through age-specific analysis, we identified high-risk age groups and examined trends over time. We also compared differences and changes between males and females.

### Statistical Analysis

The data were extracted from the Health Insurance Review and Assessment Service big data system. Analysis was performed at Wonju data center, and the SAS 9.4 program (SAS Institute, Cary, NC, USA) was used for statistical analysis. The data were analyzed using descriptive statistics.

## 3. Results

### 3.1. Changes in Prevalence of Each STI

**Figure 1:**
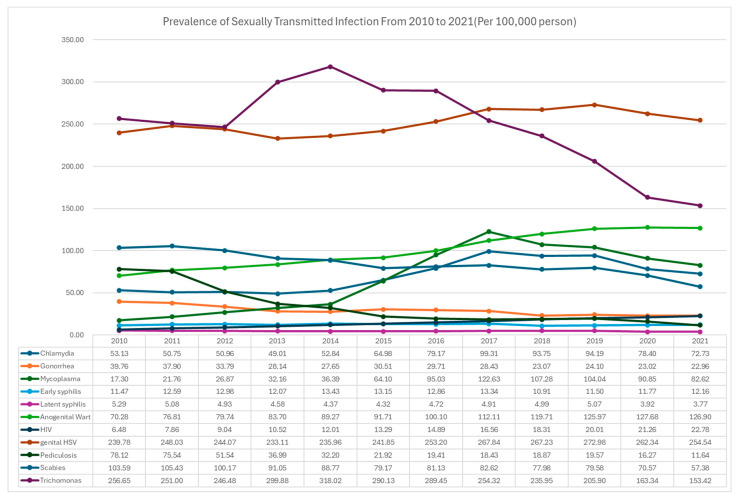
Changes in prevalence of sexually transmitted infection from 2010 to 2021 in Korea.

Among the pathogen-specific classifications, the STI with the highest prevalence in 2010 was trichomonas (256.65 per 100,000 persons), while the STI with the lowest prevalence was latent syphilis (5.29 per 100,000 persons). In 2021, the STI with the highest prevalence was genital HSV (254.54 per 100,000 persons) (Figure 1).

Overall, bacterial STIs showed a clear decreasing trend in recent years, whereas viral STIs showed a continuous increasing trend. Protozoa and parasite STIs also showed a decreasing trend.

### 3.2. Bacterial STIs

**Figure 2:**
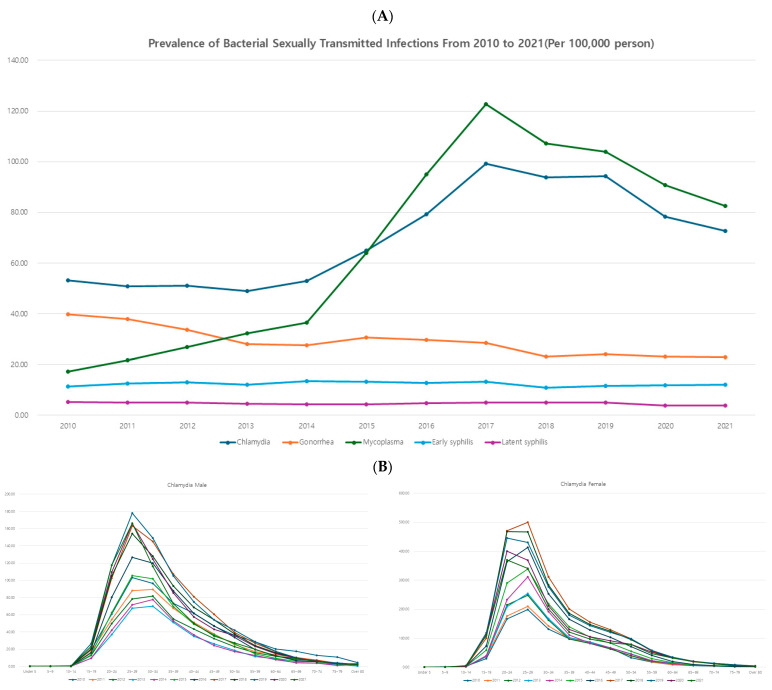

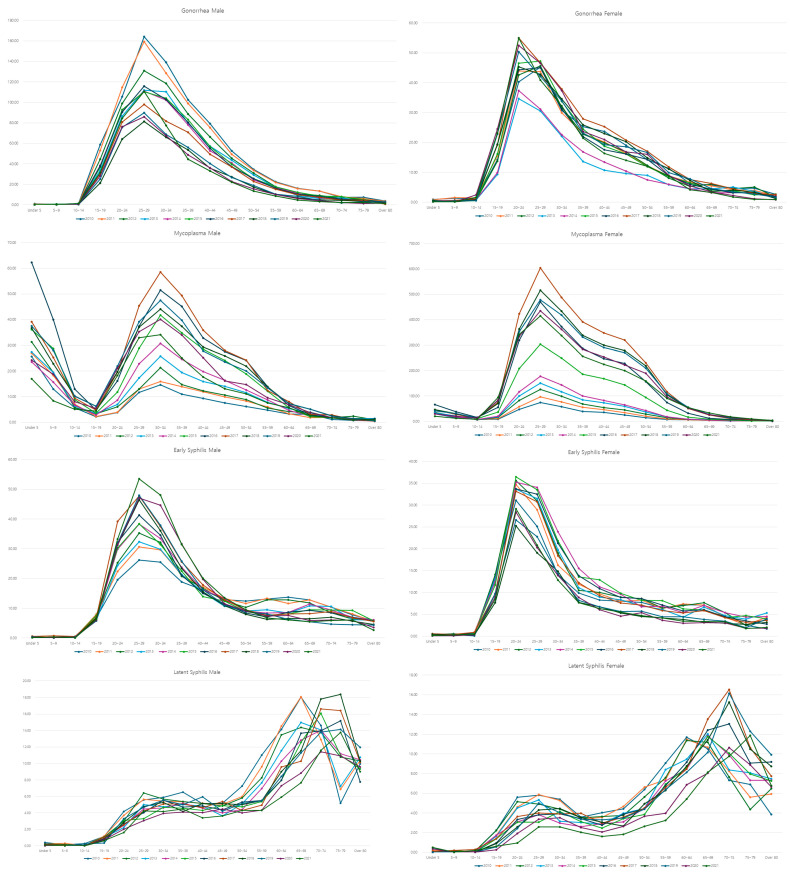
(**A**) Prevalence of each sexually transmitted infection—bacterial infection. (**B**) Age-specific prevalence of each STI—bacterial infection.

Overall, bacterial STIs showed a clear decreasing trend in recent years. Chlamydia and mycoplasma showed a steady increase until 2017, after which they exhibited a decreasing trend. Gonorrhea demonstrated a continuous decline throughout the study period. Among the types of syphilis, early syphilis did not show significant changes during the study period, while latent syphilis showed a decreasing trend (Figure 2A).

When examining the age-specific prevalence rates in 5-year intervals, chlamydia, gonorrhea, early syphilis, and female mycoplasma showed a single peak pattern at ages 20–24 or 25–29. Male mycoplasma exhibited an additional peak in early childhood, resulting in a bimodal peak pattern. Latent syphilis displayed a minor peak in the sexually active age group for both genders and a high peak in the elderly, with prevalence increasing with age (Figure 2B).

### 3.3. Viral STI

**Figure 3:**
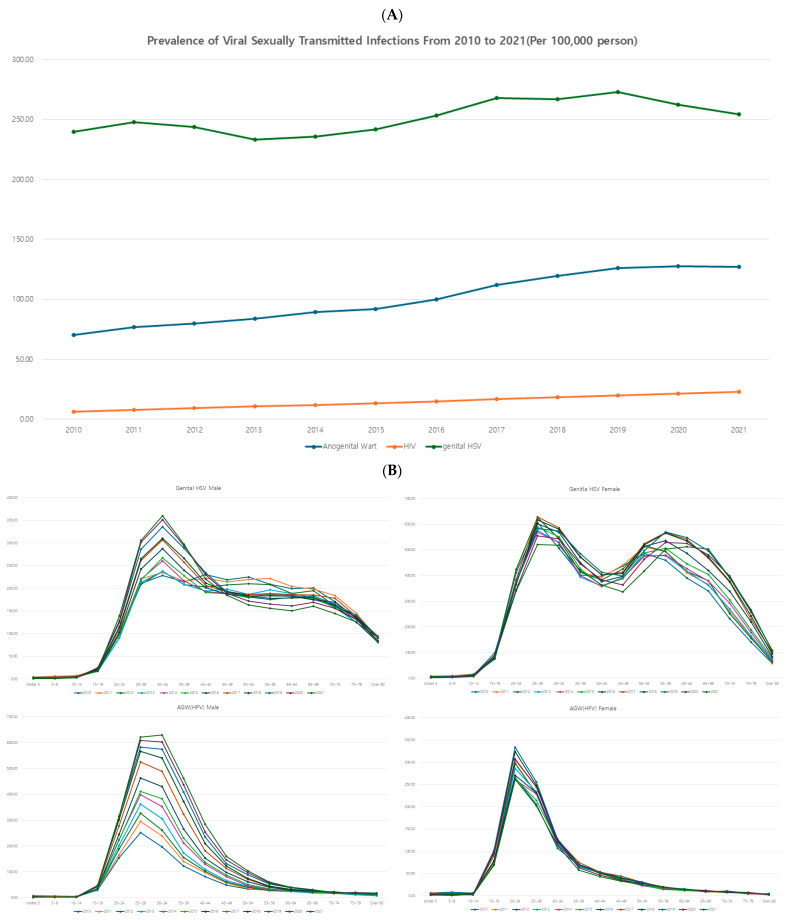

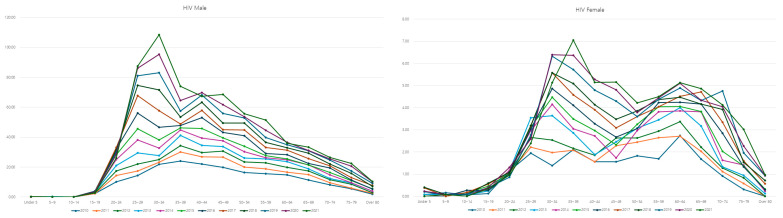
(**A**) Prevalence of each sexually transmitted infection—viral infection. (**B**) Age-specific prevalence of each STI—viral infection.

Overall, viral STIs showed a continuous increasing trend. Among viral STIs, HSV was the most common, followed by AGW and HIV. Viral STIs showed a continuous increasing trend throughout the study period (Figure 3A).

When examining age-specific prevalence rates in 5-year intervals, AGW exhibited a single high peak at ages 25–29 for men and 20–24 for women, both of which are sexually active age groups. HSV and HIV showed a main peak in the sexually active age group and a minor peak in older ages (Figure 3B).

### 3.4. Others (Parasitic and Protozoal)

**Figure 4:**
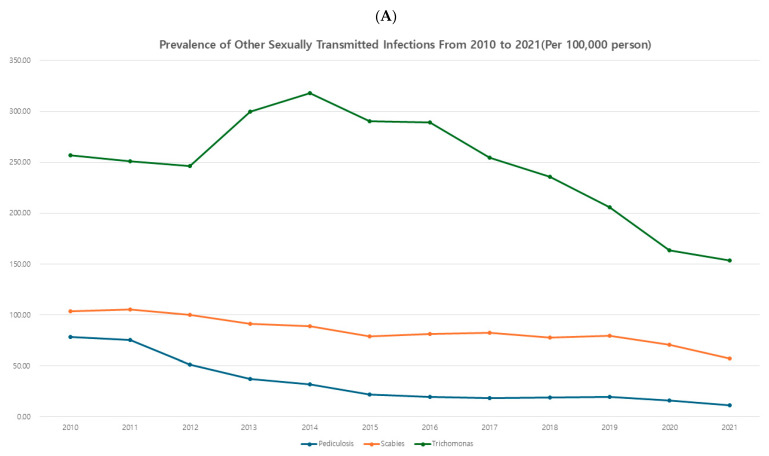

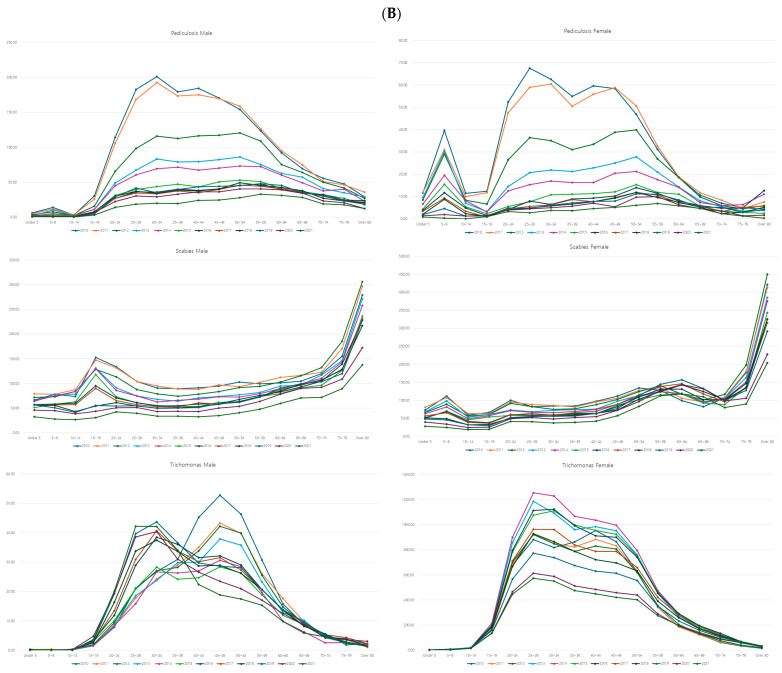
(**A**) Prevalence of each sexually transmitted infection—other infection (protozoa and parasite). (**B**) Age-specific prevalence of each STI—other infection (protozoa and parasite).

Overall, protozoa and parasite STIs also showed a decreasing trend. Scabies and pediculosis showed a continuous decline, while trichomonas peaked in 2014 and subsequently exhibited a continuous decrease (Figure 4A).

In age-specific prevalence rates by 5-year intervals, scabies increased in the elderly and showed a minor peak in males aged 15–29 and in females around ages 5–9. Pediculosis exhibited a relatively broad peak distribution compared to other pathogens analyzed, with a minor peak in both genders at ages 5–9. The protozoa infection trichomonas showed an initial peak in males aged 40–50, which shifted to younger ages over time, peaking at ages 25–34. In females, trichomonas showed a broad peak distribution from ages 20–54 (Figure 4B).

### 3.5. Non-Gonococcal Urethritis

**Figure 5:**
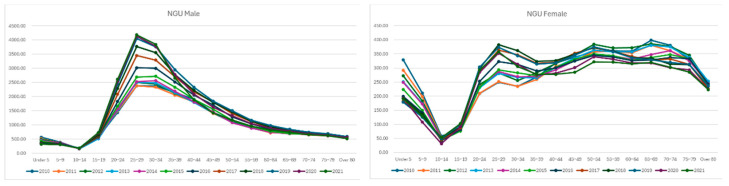
Age-specific prevalence of each STI—Non-gonococcal urethritis.

NGU showed a continuous increase until 2019, followed by a slight decrease after 2020. In terms of prevalence, it was the most common STI diagnosis. In males, there was a major peak at ages 25–29, with a relatively broad distribution across ages 20–45. In females, the prevalence exhibited a “√” shape, with a low point in the 10–19 age group, and then maintained a consistent level across all other age groups, extending into older ages (Figure 5).

### 3.6. Difference Between Males and Females

**Figure 6:**
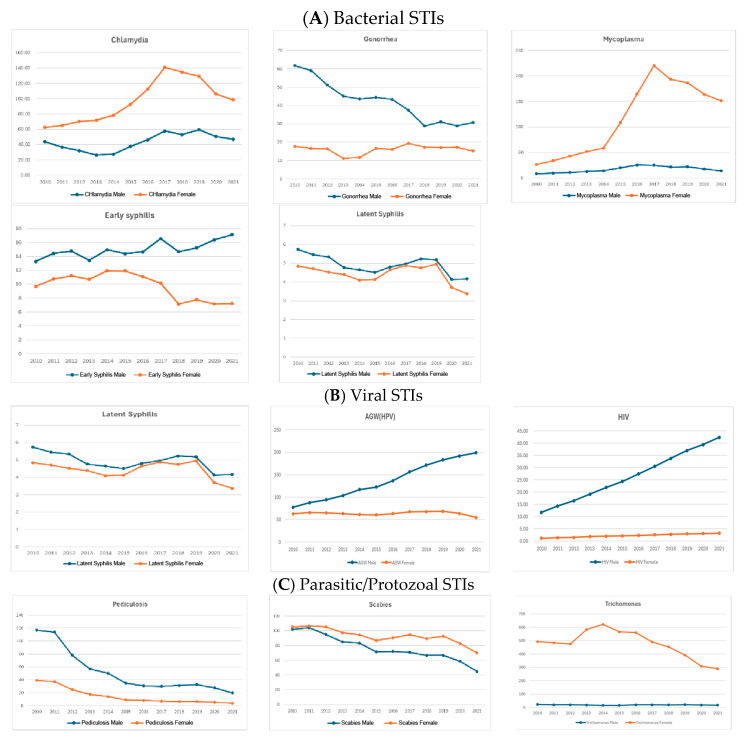
Changes in prevalence between males and females from 2010 to 2021.

The prevalence rates varied by gender for different pathogens. Gonorrhea, early and latent syphilis, AGW, HIV, pediculosis, and NGU were more common in males, while chlamydia, mycoplasma, genital HSV, scabies, and trichomonas were more common in females (Table 1; Figure 6). The gender disparity in the prevalence of chlamydia, mycoplasma, early syphilis, AGW, and scabies has increased over time, whereas the male-to-female ratio for latent syphilis, genital HSV, and HIV has remained consistent.

## 4. Discussion

In this study, different patterns were observed compared to some studies conducted abroad. Overseas, syphilis, chlamydia, trichomoniasis, and genital herpes have shown increasing trends [1]. In the current study, the results showed that chlamydia and mycoplasma increased until 2017 and then started to decline. Syphilis and gonorrhea also showed a decreasing trend, while parasitic and protozoa infections continuously decreased. However, genital HSV, HIV, and AGW demonstrated a continuous increase over the period. In other words, viral STIs have been steadily increasing, while other STIs have shown a decreasing trend over the past five years. Additionally, most diseases showed the highest prevalence among sexually active age groups. However, scabies and latent syphilis, while exhibiting some peaks among the sexually active young population, showed higher prevalence primarily in the elderly. This indicates that, despite being sexually transmitted, these diseases may also have other transmission routes and characteristics that need to be understood. Moreover, while some diseases show a single peak, others have multiple peaks, necessitating different approaches based on the prevalent age groups. In terms of gender differences, gonorrhea, early syphilis, latent syphilis, AGW, HIV, pediculosis (crab lice), and NGU were more prevalent in men. Conversely, chlamydia, mycoplasma, genital HSV, scabies, and trichomoniasis were more prevalent in women. The gender gap is widening for diseases like chlamydia, mycoplasma, early syphilis, AGW, scabies, and NGU. Therefore, it is necessary to adopt different approaches based on gender, age, and time period for each disease.

According to the WHO fact sheet, the following facts about STIs are listed: (1) More than 1 million curable STIs are acquired every day worldwide in people 15–49 years old, the majority of which are asymptomatic. (2) In 2020 there were are an estimated 374 million new infections in people 15–49 years with one of four curable STIs: chlamydia, gonorrhea, syphilis, and trichomoniasis. (3) An estimated 8 million adults between 15 and 49 years old were infected with syphilis in 2022. (4) More than 500 million people aged 15–49 years are estimated to have a genital infection with HSV [11]. (5) HPV infection is associated with over 311 000 cervical cancer deaths each year [12]. (6) A total of 1.1 million pregnant women were estimated to be infected with syphilis in 2022, resulting in over 390 000 adverse birth outcomes. (7) STIs have a direct impact on sexual and reproductive health through stigmatization, infertility, cancers, and pregnancy complications and can increase the risk of HIV. (8) Drug resistance is a major threat to reducing the burden of STIs worldwide.

Using the term STI instead of STD emphasizes the importance of asymptomatic infections and asymptomatic carriers. This shift reflects advancements in diagnostic techniques such as PCR, DNA/RNA detection, and NAAT, which allow for broader identification of not only diseases but also carriers and asymptomatic infections. Consequently, this has led to an apparent increase in prevalence rates [13]. However, as diagnoses increase, the need for treatment cannot be overlooked, leading to higher healthcare costs and increased societal burden. Additionally, addressing STIs requires a comprehensive approach that includes diagnosis, treatment, prevention, and education, along with collaborative efforts from both the nation and members of society [14].

STIs are influenced by numerous factors and, in turn, affect many areas. They particularly interact with public health, healthcare policies, the flow of capital, quality of life, women’s sexual and reproductive health, culture, history, and education, among many other aspects [15,16,17]. Demographic characteristics also have an impact, and recently, STIs among the elderly due to population aging have become an important issue. There was a study conducted in Korea that concluded that STIs in the elderly were relatively stable [18]. However, as in this study, some diseases were found to be more prevalent in the elderly. Depending on the type of disease, there are diseases that require attention even in the elderly. While many sexually transmitted diseases have a long history and have been around for a long time in human history, the most recent one, monkeypox, has been classified as a sexually transmitted disease because it shows characteristics similar to sexually transmitted diseases [19,20].

The increase or endemicity of a specific sexually transmitted infection affects the surrounding countries, including the country in question. In general, some STIs are managed by including them in the sample surveillance system and statutory infectious diseases in Korea [21]. Syphilis, which has been steadily increasing every year in Japan, has recently become a major social issue [22,23]. In the United States, alongside the rise in bacterial STIs, antibiotic resistance in pathogens such as mycoplasma and gonorrhea has become a significant concern, leading to changes in recommendations in the CDC guidelines [4]. In Korea, there was also a revision to the law requiring all syphilis patients diagnosed after January 2024 to be reported. Changes in prevalence, as seen in these cases, have a significant impact on shaping health policies and modifying clinical guidelines.

This study has several limitations. First, although data extracted by main diagnosis, etc., were used, only diseases corresponding to the main diagnosis were extracted, so diseases such as concomitant infections were not included in the study. It is thought that additional analysis or research on concomitant infection rates, etc., will be necessary through additional analysis. Second, since the laboratory results were not reflected, they were extracted by diagnosis name, so actual clinical aspects or results were not included. Third, since they are diagnosis-based data, it may be judged that the scale of the disease has increased as patients who were not diagnosed due to the development of actual diagnostic techniques or diagnostic accuracy are diagnosed. However, for some diseases, such as AGW, the increase in treatment is like the increase in the disease, so it can be inferred that the change trend of the disease is related [24].

The findings of this research will contribute to the existing body of knowledge on STIs in South Korea, offering valuable information for healthcare providers, policymakers, and researchers. By identifying patterns and trends in STI prevalence, this study aims to support the development of more effective and targeted public health strategies to combat the spread of STIs and improve sexual health outcomes in South Korea.

By analyzing overall, gender-specific, and age-specific prevalence rates, this research seeks to provide insights into the current state of STIs in South Korea and identify emerging trends that may inform future public health policies. Specifically, this study will categorize STIs based on the type of pathogen—bacterial, viral, and those caused by protozoa or parasites—and examine the prevalence rates across different demographic groups. Such an approach will enable a more nuanced understanding of how STIs affect various segments of the population and highlight the need for targeted interventions to address the unique challenges posed by each type of infection. In addition, the observed trends of continuous change are expected to assist Korea’s public health authorities in determining the direction of policies related to disease prevention, education, and response, as well as in allocating appropriate budgets. Furthermore, as demonstrated in this study, the increasing trend of viral STIs may influence decisions regarding the introduction of national immunization programs. For certain bacterial STIs, the findings may also serve as valuable evidence to address issues such as antibiotic misuse, ultimately shaping future disease response strategies.

## 5. Conclusions

Bacterial STIs and other infections have shown a declining trend in recent years, whereas viral STIs have been continuously increasing. The characteristics of each pathogen vary according to age and gender, necessitating the establishment of risk groups for each pathogen and the development of prevention policies accordingly.

## Figures and Tables

**Table 1:** Male-to-female ratio of sexually transmitted disease from 2010 to 2021.

		2010	2011	2012	2013	2014	2015	2016	2017	2018	2019	2020	2021	Mean
Chlamydia	Male	1	1	1	1	1	1	1	1	1	1	1	1	
	Female	1.43	1.77	2.19	2.74	2.86	2.46	2.43	2.44	2.54	2.18	2.09	2.09	2.27
Gonorrhea	Male	1	1	1	1	1	1	1	1	1	1	1	1	
	Female	0.29	0.28	0.32	0.25	0.27	0.37	0.37	0.52	0.60	0.55	0.60	0.49	0.41
Mycoplasma	Male	1	1	1	1	1	1	1	1	1	1	1	1	
	Female	3.18	3.48	3.98	4.05	4.12	5.43	6.47	8.71	8.88	8.44	9.21	10.91	6.40
Early Syphilis	Male	1	1	1	1	1	1	1	1	1	1	1	1	
	Female	0.73	0.74	0.76	0.80	0.80	0.83	0.76	0.61	0.49	0.51	0.44	0.42	0.66
Latent Syphilis	Male	1	1	1	1	1	1	1	1	1	1	1	1	
	Female	0.84	0.86	0.85	0.92	0.88	0.92	0.97	0.98	0.91	0.96	0.90	0.81	0.90
Genital HSV	Male	1	1	1	1	1	1	1	1	1	1	1	1	
	Female	2.14	2.20	2.30	2.26	2.24	2.30	2.32	2.34	2.32	2.23	2.13	2.08	2.24
AGW	Male	1	1	1	1	1	1	1	1	1	1	1	1	
	Female	0.81	0.75	0.69	0.61	0.52	0.49	0.46	0.43	0.40	0.37	0.33	0.27	0.51
HIV	Male	1	1	1	1	1	1	1	1	1	1	1	1	
	Female	0.10	0.10	0.09	0.10	0.09	0.09	0.08	0.08	0.08	0.08	0.08	0.08	0.09
Pediculosis	Male	1	1	1	1	1	1	1	1	1	1	1	1	
	Female	0.33	0.33	0.32	0.30	0.28	0.26	0.26	0.23	0.20	0.19	0.18	0.19	0.26
Scabies	Male	1	1	1	1	1	1	1	1	1	1	1	1	
	Female	1.03	1.02	1.11	1.15	1.14	1.22	1.26	1.34	1.34	1.39	1.42	1.56	1.25
Trichomonas	Male	1	1	1	1	1	1	1	1	1	1	1	1	
	Female	21.96	24.79	24.06	32.32	39.34	35.56	28.61	25.04	23.65	18.88	16.79	16.69	25.64
NGU	Male	1	1	1	1	1	1	1	1	1	1	1	1	
	Female	0.21	0.21	0.22	0.22	0.21	0.20	0.19	0.18	0.18	0.17	0.16	0.17	0.19

## Data Availability

The excel data used to support the findings of this study were supplied by Sangrak Bae under license, and requests for access to these data should be made to Sangrak Bae robinbae97@gmail.com.
